# Methodological Considerations in Interpreting Mortality Trends in Aortic Dissection Among Hypertensive Adults

**DOI:** 10.1002/clc.70340

**Published:** 2026-05-04

**Authors:** Hamna Bibi, Saad Arif

**Affiliations:** ^1^ Ayub Medical College Abbottabad Pakistan

Dear Editor,

We read with great interest the recent nationwide analysis examining mortality due to aortic dissection (AD) among adults with primary hypertension over two decades. The study provides valuable population level data describing temporal and demographic trends [1]. However, several methodological considerations require clarification to prevent potential overinterpretation of the findings. Clarification on these points would support more accurate understanding of what the data show.

Uncertainty arises when analyses rely on death certificate data, as misclassification of the underlying cause of death is common in cardiovascular conditions [2]. Both hypertension and aortic dissection may be underreported or inconsistently coded, and temporal changes in coding practices may influence observed mortality trends.

Secondly, attributing changes in mortality rates solely to improvement in blood pressure management may be misleading. Without individual data on how severe hypertension was, or whether it was treated or controlled, conclusions remain uncertain. Population level trends cannot establish that individual level hypertension management drove mortality changes [3]. National survey data indicate that improvements in blood pressure control were modest and later plateaued during the study period, suggesting that factors such as advances in imaging, emergency diagnosis, surgical intervention, or regional healthcare access may also contribute substantially to mortality patterns.

Also, defining hypertensive cohort exclusively using ICD‐10 code I10 (essential hypertension) excludes patients with hypertensive hearts or renal disease (I11‐I13) and secondary hypertension (I15). Restricting the cohort to essential hypertension may exclude patients with hypertension related and end organ damage, potentially omitting individuals at higher risk and limiting how accurately the findings reflect overall population vulnerability [4]. Consequently, the true disease burden may be underestimated.

Finally, comparison with an “AD without hypertension” group raises concerns since most people with AD have hypertension [5]. The remaining group likely includes undiagnosed hypertensives and patients with distinct etiologies such as connective tissue disorder resulting in non‐exchangeable comparison groups and limiting casual inference regarding hypertension's contribution to mortality differences.

Clarifying these boundaries is important to ensure that the study's valuable descriptive findings are not interpreted as evidence of casual mechanisms or direct effects of hypertension control alone. We appreciate the authors for their important contribution and hope this discussion supports careful interpretations and guides future work aimed at improving outcomes in aortic dissection.

## Author Contributions

All authors have read and approved the final manuscript.

## Funding

The authors have nothing to report.

## Ethics Statement

The authors have nothing to report.

## Conflicts of Interest

The authors declare no conflicts of interest.

## Data Availability

The data used in this study is publicly available. No new data were generated.
